# Prenatal Exposure to Environmental Tobacco Smoke and Hyperactivity Behavior in Chinese Young Children

**DOI:** 10.3390/ijerph14101132

**Published:** 2017-09-27

**Authors:** Qingmei Lin, Xiang-Yu Hou, Xiao-Na Yin, Guo-Min Wen, Dengli Sun, Dan-Xia Xian, Lijun Fan, Hui Jiang, Jin Jing, Yu Jin, Chuan-An Wu, Wei-Qing Chen

**Affiliations:** 1 School of Public Health, Sun Yat-sen University, Guangzhou 510080, China; linqm01@163.com (Q.L.); fanlijun3@mail.sysu.edu.cn (L.F.); jiangh43@163.com (H.J.); jingjin@mail.sysu.edu.cn (J.J.); jinyu@mail.sysu.edu.cn (Y.J.); 2 Australia China Centre for Public Health, Brisbane, Queensland 4059, Australia; x.hou@qut.edu.au; 3 School of Public Health and Social Work, Queensland University of Technology, Brisbane, Queensland 4059, Australia; 4 Maternal and Child Health Care Center of Longhua New District, Shenzhen 518131, China; yinxn1211@163.com (X.-N.Y.); 13923453902@163.com (G.-M.W.); sunml627@163.com (D.S.); xiandanxia@163.com (D.-X.X.)

**Keywords:** environmental tobacco smoke, prenatal, hyperactivity behavior, children

## Abstract

This study aimed to examine the association between prenatal environmental tobacco smoke (ETS) exposure and hyperactivity behaviors in young children. A cross-sectional study was undertaken among 21,243 participants from all of the kindergartens in Longhua District of Shenzhen, China. Multivariate logistic regression models and hierarchical linear models were employed to assess the associations. After adjusting for potential confounders of gender, preterm birth, birth asphyxiation, etc., prenatal ETS exposure was significantly associated with an increased risk of hyperactivity behaviors in young children (OR (95% CI) = 1.51 (1.28–1.77); β (95% CI) = 0.017 (0.013–0.020)). Along with increases in children’s prenatal ETS exposure dose (measured by daily ETS exposure duration, daily cigarette consumption by household members, and overall score of prenatal ETS exposure), the children were also increasingly more likely to exhibit hyperactivity behaviors. Furthermore, children whose mothers had prenatal ETS exposure in any one or more of the pregnancy trimesters were more likely to exhibit hyperactivity behaviors as compared with those born to non-exposure mothers (all *p* < 0.05). Overall, prenatal ETS exposure could be associated with a detrimental impact on offspring’s hyperactivity behaviors, and public health efforts are needed to reduce prenatal ETS exposure.

## 1. Introduction

Hyperactivity behaviors are the main clinical manifestation of attention-deficit hyperactivity disorder and belong to one of the most common neurobehavioral conditions in preschool and school children [1,2], which are likely to disrupt their academic achievements, social relationship establishment, and occupational functioning [3]. The cause of hyperactivity behaviors remains unclear, but a series of prior epidemiological evidence have identified a relationship between them with prenatal exposure to harmful environmental factors, among which the impact of maternal active smoking on offspring behaviors is quite well-established [4,5,6]. For instance, Linnet et al. reported that smoking in pregnancy increased the prevalence of hyperkinetic disorder by threefold [7]. Another study [8] investigated 3.5-year-old preschool children and observed that when compared with children of non-smokers, children born to mothers who smoked 10 or more cigarettes per day had a 60% increased risk of hyperactivity and distractibility. Based on a large, nationally representative sample of children aged 4–11 years, Weitzman et al. [9] suggested an independent relationship between maternal smoking both during and after pregnancy and the increased rates of behavioral problems in children, and their further evidence indicated a dose-response relationship. Besides, animal studies have also shown that exposure to nicotine in utero is associated with hyperactivity in the offspring [10].

It has been well documented that prenatal exposure to environmental tobacco smoke (ETS) has similar effects of maternal active smoking on birth outcome and child health [11,12]. However, its impact on hyperactivity behaviors in the offspring has been less studied despite the large number of exposed pregnant women [13], and there remains much controversy regarding their actual association. A few studies found that fetus exposure to ETS was related to a higher risk of aggression and externalizing behavior problems [14,15,16], and one study in China also documented similar results [17]. Furthermore, in a prospective birth cohort study, Ruckinger et al. [18] investigated the impact of ETS exposure at home during either the prenatal or postnatal period, or both periods, and reported an increased risk of behavioral problems in 10-year-old children for those with intrauterine and postnatal exposures. Tiesler et al. [19] also found that the child was at an increased risk of developing hyperactivity/inattention problems when their non-smoking mother was exposed prenatally and postnatally to cigarette smoke of the father or other household members. Nevertheless, there were some other studies [20,21,22] reporting that paternal smoking during the maternal pregnancy period was not significantly related to offspring hyperactivity. Most of such studies came from European countries, where the maternal active smoking rate was usually high, the children’s main age was 10 years, and the sample size was no more than 6000 children.

On the whole, previous research focused more on maternal active smoking rather than ETS exposure and had inconclusive findings regarding the impact of prenatal exposure to ETS; even the scarce amount of research on ETS was limited in western countries, representing relatively old children or small sample sizes. Further evidence is warranted to investigate the more exact association between maternal ETS exposure during pregnancy and child behaviors, especially in younger children and in countries like China, where less than 2% of women smoked [23] but approximately 42% of women were exposed to ETS during pregnancy [24]. Thus, this study, based on a large sample in Shenzhen, China, aimed to assess the association of maternal ETS exposure during pregnancy and trimester-specific ETS exposure with offspring’s hyperactivity behaviors in early life, as well as a dose-response relationship between them.

## 2. Materials and Methods

### 2.1. Participants

The Longhua Child Cohort Study (LCCS) is an on-going population-based child cohort study (starting from September 2014 and following up once per year) to assess the influences of family and school environment surrounding children’s early life on child psycho-behavioral development. In the LCCS, all of the children were enrolled in the study at their first entrance into kindergartens in the Longhua District of Shenzhen, China, and the primary caregivers of children were contacted to fulfill a self-administered structured questionnaire. Participants for this study were recruited from the two baseline surveys of the LCCS taken place in 2014 and 2015, during when a total of 25,070 children were approached. After excluding children whose mothers were active smokers before or during pregnancy and those who refused to respond or with incomplete information on exposures and outcomes, the remaining 21,243 (84.7% in 25,070) children were included for analysis in this study. Among the included questionnaires, 87.7% were completed by mothers of the children, 9.7% by fathers, and 2.6% by others (including grandparents etc.). All of the subjects gave their informed consent for inclusion before they participated in the study. The study was conducted in accordance with the Declaration of Helsinki, and the protocol was approved by the Ethics Committee of the School of Public Health at Sun Yat-sen University (ethics clearance No.: 2015–016).

### 2.2. Data Collection

Data were gathered through structured questionnaires, which contained information about parents’ socio-demographic characteristics (age, education, marital status, and family income) and children’s general information including date of birth, gender, single child or not, preterm (gestation < 36 weeks), low birth weight (the infant with birth weight ≤ 2500 g), prenatal ETS exposure in different time periods of pregnancy, and the child’s hyperactivity behavioral problems.

### 2.3. Measurements

#### 2.3.1. Measurement of Environmental Tobacco Smoke (ETS) Exposure

Maternal exposure to ETS during pregnancy was assessed by the following questions: (1) Did any household members of the pregnant mother ever smoked at home (0 score = ‘no’, 1 = ‘yes’); and if yes: (2) How many cigarettes did they smoked per day at home (1 score = ‘1–5 cigarettes’, 2 = ‘6–10 cigarettes’, 3 = ‘11–15 cigarettes’, 4 = ‘>15 cigarettes’); and (3) How long was the pregnant mother exposed to ETS per day (1 score = ‘<15 min’, 2 = ‘15–30 min’, 3 = ‘>30 min’). Thereafter, we calculated an ETS exposure score by multiplying the answers to the latter two questions concerning the number of cigarettes consumed by smokers and the duration of mothers’ prenatal ETS exposure per day at home (where a higher score showed a higher ETS exposure level), and this ETS exposure score was additionally grouped into four categories (i.e., ‘0 score’, ‘1 score’, ‘2–3 score’, and ‘>3 score’) according to the distribution of each score’s percentage of the whole. We further collected information regarding trimester-specific tobacco smoke exposure by asking whether the pregnant mother was ever exposed to tobacco smoke at home during each of the three pregnancy trimesters (‘yes’ or ‘no’). Children were then classified into nine categories according to the mother’s ETS exposure during each trimester.

#### 2.3.2. Measurement of Hyperactivity Behaviors

Children’s hyperactivity behaviors were measured by the hyperactivity index (HI) subscale in Conners’ Parent Rating Scale-Revised (CPRS-48), an internationally disseminated and validated screening tool to assess behavioral difficulties in children aged between 3–16 years old [25]. This tool had been translated into Chinese and showed a good reliability and validity as well [26]. The HI measure is comprised of 10 items, and each item is rated on a 0 to 3 scale depending on the extent to which each statement is true of the children’s behavior, i.e., never (for a score of 0), sometimes (score of 1), often (score of 2), and frequently (score of 3). The average score was then calculated, with a maximum possible value of 3. The measurement of hyperactivity behaviors was originally a continuous variable ranging between 0–3, where a higher score indicated a higher level of hyperactivity behaviors. It was also treated categorically in previous literature, using a cut-off score of 1.5 to identify the children with and without hyperactivity behaviors [27]. In the current study, the measurement of hyperactivity behaviors was treated in both categorical and continuous formats for analysis.

#### 2.3.3. Confounding Variables

The following confounding covariates were chosen based on the published literature (which previously had known effects), including child age, gender, preterm birth, low birth weight, birth asphyxia, fetal growth restriction, single child or not, mode of delivery, family income, parents’ marital status, education level, and parental age at childbirth. These information were reported by the primary caregivers of children who fulfilled the study questionnaire. Specifically, a low birth weight was reported to the closest 0.5 kg, and the weight being less than 2.5 kg was regarded as low birth weight; preterm birth, birth asphyxia, fetal growth restriction, and single child were reported as yes/no; and mode of delivery was either natural labor or cesarean section. The variables with *p* < 0.1 in univariate analysis were entered in the multivariate models to control for their potential confounding effect; and those otherwise (*p* ≥ 0.1, including age, low birth weight, single child, mode of delivery, and parental marriage status) were excluded for inclusion in the multiple models.

### 2.4. Data Analysis

The socio-demographic characteristics between children with and without hyperactivity traits were compared using chi-square test for categorical variables and t-test for continuous variables. The association between ETS and hyperactivity behaviors was examined using a series of logistic regression models (when the measurement of hyperactivity behaviors took a categorical format) and hierarchical linear models (when the measurement of hyperactivity behaviors took a continuous format). In particular, given that the outcome variable (i.e., hyperactivity behaviors; when it was used in a continuous format) was not normally distributed and that it had a large number of zero values, we thus obtained a new hyperactivity score by adding “1” to the original score (to avoid zero values), and then log transformed the new score (to reduce skew) for use in the linear regression models. Odds ratio (OR) and 95% confidence interval (95% CI), or β, and 95% CI were presented as appropriate to show the strength of association. The exact formats of models are shown in the equations below:

(i) Logistic regression—when Y was categorical (Y was coded ‘0’ or ‘1’):
  Logit (p) = Log [p/(1 − p)] = β_0_ + β_1_X_1_ + β_2_ X_2_ + β_3_ X_3_ +…+ β_k_ X_k_ + ε_i_
(ii) Linear regression—when Y was continuous (0 ≤ Y ≤ 3):
  Log (Y + 1) = β_0_ + β_1_X_1_ + β_2_ X_2_ + β_3_ X_3_ +…+ β_k_ X_k_ + ε_i_

where the outcome variable (Y) is the measurement of hyperactivity behaviors described earlier. p is the probability that the event Y occurs. X_1_ is the explanatory variable of interest (i.e., ETS exposure). X_2_, X_3_, …, X_k_ represent a series of confounders including gender, preterm birth, birth asphyxiation, fetal growth restriction, family income, paternal and maternal education, and paternal and maternal age at childbirth. In this study, X_2_, X_3_, …, X_k_ were excluded in unadjusted models (Model 1: Models that were unadjusted for any confounding variables), but were included in adjusted models (Model 2: models that were adjusted for confounders). βs are the regression coefficients of the covariates, and ε_i_ is a vector of unknown residuals.

All of the analyses were carried out using the statistical software SPSS 20 (IBM, Armonk, NY, USA). Statistical analyses were two-tailed, and *p* < 0.05 was considered statistically significant.

## 3. Results

Of the 21,243 children included in this study, 686 (3.23%) were identified as having hyperactivity traits within the borderline/clinical range. The participating children had an average age of about three years (mean ± SD: 3.36 ± 0.35 years, range: 1.3–5.7 years), and approximately half of them were boys (male: 54.6%, female: 45.4%). Table 1 presents the socio-demographic characteristics of children. Between the children with and without hyperactivity behaviors, significant differences were observed in terms of child gender, preterm birth, birth asphyxia, fetal growth restriction, family income per month, parent’s educational level, and parental age at childbirth; however, other characteristics were quite comparable, including child age, low birth weight, single child, delivery mode, and parental marital status.

The associations between prenatal ETS exposure and hyperactivity behaviors in young children are presented in Table 2. In the univariate analyses, prenatal ETS exposure was significantly associated with hyperactivity behaviors in young children, and a greater dose in ETS exposure was all significantly related to a higher risk. After adjusting for the potential confounders including child’s gender, preterm birth, birth asphyxiation, fetal growth restriction, family income, parental education, and parental age during pregnancy, when compared with non-exposed children, those with prenatal exposure to ETS was still significantly associated with an increased risk for offspring hyperactivity behaviors (OR = 1.51, 95% CI = 1.28–1.77, *p* < 0.001; β = 0.017, 95% CI = 0.013–0.020, *p* < 0.001). Both the increased duration of prenatal ETS exposure and the increased number of cigarettes exposed to daily in the household were related with a higher risk (*p* < 0.05). Additionally, further dose-response analysis examining a more comprehensive indicator of the extent of ETS exposure amount (i.e., the score of prenatal ETS exposure at home) revealed that a higher overall score (meaning a higher ETS exposure level) was associated with a greater hyperactivity risk for children (adjusted OR ranged from 1.22 to 2.48, and adjusted β increased from 0.010 to 0.032).

Specifically, for the hierarchical linear models in Table 2 (last column), changes in the adjusted R square from a model that only included covariates to a model that included both covariates and the variables of interest (i.e., ETS exposure variable) were as follows: (1) prenatal ETS exposure at home: increased from 0.025 to 0.030; (2) duration of prenatal ETS exposure daily at home: increased from 0.025 to 0.031; (3) the number of cigarettes consumed by smokers daily at home: increased from 0.025 to 0.031; and, (4) the score of prenatal ETS exposure at home: increased from 0.025 to 0.032, and the F test indicated statistical significance for all of the R square changes. Effect sizes (95% CI) for the above four models were 0.005 (0.003–0.007), 0.006 (0.004–0.008), 0.006 (0.004–0.008), and 0.007 (0.004–0.009), respectively.

Furthermore, we assessed the association of child hyperactivity behaviors with combinations of prenatal ETS exposure in different trimesters of maternal pregnancy, and the results are shown in Table 3. When compared with non-exposure to ETS in all of the three pregnancy trimesters, prenatal exposure to ETS in any one or more of the trimesters was significantly associated with a higher risk of hyperactivity behaviors for young children, in both models that were unadjusted or adjusted for the aforementioned confounders. Specifically, for the hierarchical linear models in Table 3 (last column), the adjusted R square increased from 0.025 to 0.033, when the model changed from one that only included covariates to one that included both covariates and the variables of interest (i.e., ETS exposure variable); and the F test indicated statistical significance for the R square change. Effect size (95% CI) for the model was 0.008 (0.006–0.011).

## 4. Discussion

Based on large-scale baseline surveys (21,243 children) from the Longhua Child Cohort Study in Shenzhen of China, we assessed the associations between prenatal ETS exposure and the child’s early-onset hyperactivity behaviors. To the best of our knowledge, this is the first study that has considered the impact of prenatal ETS exposure in young children by using a large population-based sample, and this study is novel and meaningful in that it has explored the dose-response relationship between maternal exposure to ETS during pregnancy and a child's hyperactivity behaviors, as well as the trimester-specific influences of prenatal ETS exposure.

A range of previous research focused on the association of maternal active and passive smoking with child neurobehavioral development. For example, a prospective study in Germany supported an association between maternal smoking during pregnancy and hyperactivity or inattention problems in about 1600 10-year-old children [19]. One study [17] in China reported that maternal ETS exposure was associated with a higher risk of externalizing behavior problems in offspring aged 5–6 years old (OR = 2.08, 95% CI = 1.27–3.43). A recent study in South Korea [28] also demonstrated that the risk of attention deficit hyperactivity disorder (ADHD) was 1.17 times higher (95% CI: 1.39–1.98) if the father smoked during the mother’s pregnancy. Notably, one study across six European countries [22] documented that the association of maternal smoking with offspring ADHD was stronger than that of paternal smoking during pregnancy. Keyes et al. [21] demonstrated that maternal smoking during gestation was associated with offspring hyperactivity; moreover, they observed an increase in the extent of relationship among those smoking 10–19 and 20+ cigarettes per day. These studies reported a negative impact of prenatal smoking exposure on offspring’s neurobehavioral development; however, there were also some inconsistent findings from previous research, reporting that maternal secondhand smoking during pregnancy period was not significantly related to offspring hyperactivity [20,21,22]. 

In our present study, we observed that maternal ETS exposure during pregnancy was significantly associated with an increased risk of hyperactivity behaviors in young children. Further results showed that increases in the daily ETS exposure duration, the daily cigarette consumption by household members, and the overall score of prenatal ETS exposure, were all associated with a higher risk of children’s hyperactivity behaviors, which therefore provided some support for a dose-response relationship between prenatal ETS exposure and child's hyperactivity behaviors.

The potential mechanism for the observed associations found in our study, as well as some prior research, may be related to the neurotoxic chemical compounds of tobacco smoke, especially nicotine. It is speculated that nicotine could be transferred from mother to fetus via the placental barrier [29] and might act as a neuroteratogen on the developing brain (possibly through stimulating the nicotinic acetylcholine receptors, which then may directly perturb critical cell growth processes controlled by acetylcholine or may affect catecholaminergic neurotransmitter systems) to cause behavioral problems [30,31,32,33]. However, there were still discrepancies in findings, which might be owing to the different investigated populations, the cultural and socioeconomic variations across countries, and the varied definitions in ETS across studies.

Furthermore, in order to find out the critical sensitive periods during maternal pregnancy, our study explored the impact of trimester-specific ETS exposure on child behaviors, and observed that children whose mothers had prenatal ETS exposure in any one or more of the pregnancy trimesters were more likely to have hyperactivity behaviors when compared with those born to non-exposed mothers. Our findings showed that each pregnancy trimester represented a possibly sensitive period for accumulating the detrimental effect from ETS exposure. The results were not fully consistent with some other studies. For example, Tiesler et al.’s study also investigated the effect of ETS exposure during specific trimesters of pregnancy [19]. They found that children exposed to maternal smoking up to the third trimester showed increased risks of externalizing problems (conduct problems and hyperactivity/inattention) at the age of 10 years. However, for exposures confined to early pregnancy, no significant effects on behavioral problems were observed. Moreover, a rat model has indicated that there is a period during the second and third trimester in which neurodevelopment is particularly susceptible to perturbation by exogenous nicotine exposure, and that these adverse effects can be avoided by limiting the exposure to early gestation [34].

The following reasons may probably account for the aforementioned inconsistencies in findings, including the differences across studies in the investigated exposure (ETS exposure inclusive of nicotine and other tobacco compounds vs. nicotine exposure only) and outcome (hyperactivity behaviors vs. other neurobehavioral problems), as well as the demographic variations of different populations. Also, we speculate on another possible explanation, which could be relating to the great many participants in this study who were unsure of their exact exposed trimesters and thus could not be accurately grouped, and the small number of cases with hyperactivity behaviors in certain subgroups of different ETS exposure status in three pregnancy trimesters; these restrictions, hence, would be likely to cause our results to be by-chance and instable, thereby hindering us from finding out the exact ETS-sensitive periods in pregnancy. However, the definite reasons still remain uncertain and warrant further investigation.

Nevertheless, this study has certain limitations. First, although the present study had a large sample size of over 20,000 subjects, they were all recruited from one single district in Shenzhen, China (a city characterized with a developed industry and a sizable proportion of floating population). This may introduce the selection bias and limit the generalization of results to all children in other different areas of China. Second, the questionnaire survey was based on the retrospective recall on maternal ETS exposure, which might result in an information bias. Third, there was no uniform definition for ETS. In our study, ETS was defined as having at least one person who smoked at home. We did not cover the information about passive smoking in the workplace, thus an underestimation of ETS exposure during pregnancy may be possible. Fourth, a sizable proportion (3795, 17.9%) of participants in this study were unsure of their exact exposed trimesters and thus could not be accurately categorized into subgroups with different ETS exposure status in three pregnancy trimesters. The inaccurate subgrouping, along with the small number of hyperactivity cases in certain subgroups, tend to make our results instable and by-chance, thus contaminating our ability in determining the exact ETS-sensitive pregnancy periods. Results based on inferential statistics for these subgroups with low frequency of hyperactivity cases should be interpreted with great caution, and further studies with larger size are needed to confirm our findings. Fifth, future birth cohort studies, including biomarkers and wearable devices to track ETS exposure in an objective manner, would be more informative.

## 5. Conclusions

This study found that prenatal ETS exposure could be associated with a detrimental impact on offspring’s hyperactivity behaviors. Policy implications from our study include that the measures taken to reduce prenatal ETS exposure could be beneficial for offspring’s neurobehavioral health, e.g., through health education provided to mothers and household members to increase their awareness of ETS avoidance as a vital component of prenatal care, initiating legislation to avoid smoking at certain places, etc. Yet, the current study might be limited in terms of its cross-sectional design, the sample selection in only one city, which might not be representative of others, the retrospective survey of exposure variable, and the lack of wearable devices and biomarkers to more objectively measure ETS exposure. Therefore, future studies that adopt birth cohort design, use multi-center samples, and employ wearable devices and biomarkers are warranted and recommended to further confirm, if any, the causal association between prenatal ETS exposure and offspring hyperactivity behaviors.

## Figures and Tables

**Table 1:** Socio-demographic characteristics of participants and their associations with hyperactivity behaviors.

Characteristics	Total, N	Hyperactivity Behaviors		χ^2^/t	*p* Value
		Yes, N (%) ^†^	No, N (%) ^†^		
Age, Mean ± SD	21,243	3.34 ± 0.37	3.36 ± 0.35	−1.035	0.301
Gender				26.85	0.000 ^c^
Male	11,598	441 (64.3)	11,157 (54.3)		
Female	9645	245 (35.7)	9400 (45.7)		
Preterm birth				5.51	0.019 ^a^
Yes	1475	63 (9.2)	1412 (6.9)		
No	19,768	623 (90.8)	19,145 (93.1)		
Low birth weight				1.63	0.20
Yes	1457	56 (8.2)	1401 (6.8)		
No	18,876	609 (88.8)	18,267 (88.9)		
Missing	910	21 (3.1)	889 (4.3)		
Birth asphyxia				5.83	0.016 ^a^
Yes	322	18 (2.6)	304 (1.5)		
No	20,921	668 (97.4)	20,253 (98.5)		
Fetal growth restriction				15.11	0.000 ^c^
Yes	349	24 (3.5)	325 (1.6)		
No	20,894	662 (96.5)	20,232 (98.4)		
Single child				0.27	0.61
Yes	12,991	426 (62.1)	12,565 (61.1)		
No	8252	260 (37.9)	7992 (38.9)		
Mode of delivery				0.05	0.83
Natural labor	11,421	372 (54.2)	11,049 (53.7)		
Cesarean section	9708	311 (45.3)	9397 (45.7)		
Missing	114	3 (0.4)	111 (0.5)		
Family monthly income				9.56	0.002 ^b^
≤10,000 RMB	9091	333 (48.5)	8758 (42.6)		
>10,000 RMB	12,152	353 (51.5)	11,799 (57.4)		
Parental marriage status				3.91	0.27
Married	20,499	656 (95.6)	19,843 (96.5)		
Remarried	227	8 (1.2)	219 (1.1)		
Divorced	249	11 (1.6)	238 (1.2)		
Widow & Unmarried	164	9 (1.3)	155 (0.8)		
Missing	104	2 (0.3)	102 (0.5)		
Paternal education				23.36	0.000 ^c^
≤Junior high school	2780	126 (18.4)	2654 (12.9)		
High school or equivalent	5290	187 (27.3)	5103 (24.8)		
≥College	13,173	373 (54.4)	12,800 (62.3)		
Maternal education				14.39	0.001 ^b^
≤Junior high school	3531	134 (19.5)	3397 (16.5)		
High school or equivalent	6453	237 (34.5)	6216 (30.2)		
≥College	11,259	315 (45.9)	10,944 (53.2)		
Paternal age at childbirth, Mean ± SD	21,243	28.68 ± 4.30	29.66 ± 4.51	5.63	0.000 ^c^
Maternal age at childbirth, Mean ± SD	21,243	26.33 ± 3.76	27.24 ± 3.85	6.05	0.000 ^c^

Notes: ^†^ All data are expressed as N (%) unless otherwise stated. ^a^ *p* < 0.05; ^b^ *p* < 0.005; ^c^ *p* < 0.001.

**Table 2:** Results from logistic and linear regression models on the association between prenatal environmental tobacco smoke (ETS) exposure and hyperactivity behaviors in young children.

Prenatal ETS Exposure	Total, N = 21,243	Hyperactivity Behaviors					
		Logistic Regression (Used as a Categorical Variable)			Linear Regression (Used as a Continuous Variable)		
		Cases, N (%)	Model 1 ^†^	Model 2 ^‡^	Score, Mean ± SE	Model 1 ^†^	Model 2 ^§^
			Crude OR (95% CI)	Adjusted OR (95% CI)		Crude β (95% CI) ^¶^	Adjusted β (95% CI) ^¶^
**Prenatal ETS exposure at home**							
No	14,533	387 (2.7)	1.00	1.00	0.613 ± 0.004	Ref	Ref
Yes	6710	299 (4.5)	1.71 (1.46–1.99) ^c^	1.51 (1.28–1.77) ^c^	0.679 ± 0.005	0.022 (0.019–0.025) ^c^	0.017 (0.013–0.020) ^c^
**Duration of prenatal ETS exposure daily at home**							
0	14,533	387 (2.7)	1.00	1.00	0.613 ± 0.004	Ref	Ref
<15 min	5247	201 (3.8)	1.46 (1.22–1.73) ^c^	1.30 (1.09–1.56) ^b^	0.663 ± 0.006	0.018 (0.014–0.021) ^c^	0.013 (0.009–0.016) ^c^
15–30 min	902	54 (6.0)	2.33 (1.74–3.12) ^c^	2.02 (1.50–2.73) ^c^	0.725 ± 0.014	0.034 (0.026–0.041) ^c^	0.028 (0.021–0.035) ^c^
>30 min	561	44 (7.8)	3.11 (2.25–4.30) ^c^	2.63 (1.89–3.66) ^c^	0.758 ± 0.018	0.041 (0.032–0.050) ^c^	0.035 (0.025–0.044) ^c^
**Number of cigarettes consumed by smokers daily at home**							
0	14,533	387 (2.7)	1.00	1.00	0.613 ± 0.004	Ref	Ref
1–5 cigarettes	3543	130 (3.7)	1.39 (1.14–1.70) ^b^	1.22 (0.99–1.50)	0.655 ± 0.007	0.017 (0.013–0.021) ^c^	0.011 (0.007–0.015) ^c^
6–10 cigarettes	1710	75 (4.4)	1.68 (1.30–2.16) ^c^	1.49 (1.15–1.93) ^b^	0.675 ± 0.010	0.020 (0.015–0.026) ^c^	0.016 (0.010–0.021) ^c^
11–15 cigarettes	726	44 (6.1)	2.36 (1.71–3.25) ^c^	2.14 (1.54–2.96) ^c^	0.738 ± 0.016	0.035 (0.027–0.043) ^c^	0.031 (0.023–0.039) ^c^
>15 cigarettes	731	50 (6.8)	2.68 (1.98–3.64) ^c^	2.39 (1.75–3.26) ^c^	0.742 ± 0.016	0.036 (0.028–0.044) ^c^	0.031 (0.023–0.039) ^c^
**The score of prenatal ETS exposure at home**							
0	14,533	387 (2.7)	1.00	1.00	0.613 ± 0.004	Ref	Ref
1	3170	115 (3.6)	1.38 (1.11–1.70) ^b^	1.22 (0.98–1.51)	0.650 ± 0.008	0.015 (0.011–0.019) ^c^	0.010 (0.006–0.014) ^c^
2–3	2048	77 (3.8)	1.43 (1.11–1.83) ^b^	1.28 (0.99–1.65)	0.674 ± 0.009	0.021 (0.016–0.026) ^c^	0.016 (0.011–0.021) ^c^
>3	1492	107 (7.2)	2.82 (2.26–3.52) ^c^	2.48 (1.98–3.12) ^c^	0.745 ± 0.011	0.037 (0.032–0.043) ^c^	0.032 (0.026–0.038) ^c^

Notes: ^†^ Model was unadjusted for any confounding variables. ^‡^ Multiple logistic regression model was used, while adjusting for gender, preterm birth, birth asphyxiation, fetal growth restriction, family income, paternal and maternal education, paternal and maternal age at childbirth. **^§^** Hierarchical linear regression model was used, while adjusting for gender, preterm birth, birth asphyxiation, fetal growth restriction, family income, paternal and maternal education, paternal and maternal age at childbirth. **^¶^**β was the coefficient for ETS exposure variable in the hierarchical linear regression model, where the dependent variable took the form of log (hyperactivity score + 1). ^b^ *p* < 0.005; ^c^ *p* < 0.001.

**Table 3:** Results from logistic and linear regression models on the association between hyperactivity behaviors of young children and combinations of prenatal environmental tobacco smoke (ETS) exposure in different trimesters of pregnancy.

Prenatal ETS Exposure			Total, N	Hyperactivity Behaviors					
1st Trimester	2nd Trimester	3rd Trimester		Logistic Regression (Used as a Categorical Variable)			Linear Regression (Used as a Continuous Variable)		
				Cases, N (%)	Model 1 ^†^	Model 2 ^‡^	Score, Mean ± SE	Model 1 ^†^	Model 2 ^§^
					Crude OR (95% CI)	Adjusted OR (95% CI)		Crude β (95% CI) ^¶^	Adjusted β (95% CI) ^¶^
No	No	No	14,533	387 (2.7)	1.00	1.00	0.613 ± 0.004	Ref	Ref
Yes	No	No	401	186 (4.0)	1.52 (0.91–2.53)	1.33 (0.80–2.23)	0.697 ± 0.021	0.029 (0.018–0.039) ^c^	0.023 (0.012–0.034) ^c^
No	Yes	No	130	9 (6.9)	2.72 (1.37–5.39) ^b^	2.36 (1.19–4.71) ^a^	0.741 ± 0.037	0.040 (0.021–0.059) ^c^	0.033 (0.014–0.051) ^c^
No	No	Yes	190	10 (5.3)	2.03 (1.07–3.87) ^a^	1.81 (0.95–3.46)	0.774 ± 0.030	0.046 (0.030–0.061) ^c^	0.040 (0.024–0.055) ^c^
Yes	Yes	No	107	8 (7.5)	2.95 (1.43–6.11) ^b^	2.43 (1.17–5.07) ^a^	0.819 ± 0.040	0.059 (0.038–0.080) ^c^	0.050 (0.029–0.070) ^c^
Yes	No	Yes	36	4 (11.1)	4.57 (1.61–12.98) ^b^	3.78 (1.31–10.88) ^a^	0.853 ± 0.070	0.067 (0.031–0.102) ^c^	0.057 (0.022–0.092) ^b^
No	Yes	Yes	202	12 (5.9)	2.31 (1.28–4.17) ^a^	1.99 (1.10–3.61) ^a^	0.679 ± 0.029	0.025 (0.010–0.040) ^b^	0.017 (0.002–0.032) ^a^
Yes	Yes	Yes	1849	105 (5.7)	2.20 (1.76–2.75) ^c^	1.93 (1.53–2.42) ^c^	0.719 ± 0.010	0.033 (0.027–0.038) ^c^	0.027 (0.022–0.032) ^c^
Unsure of the exposure trimester			3795	135 (3.6)	1.31 (1.11–1.65) ^b^	1.21 (0.98–1.48)	0.645 ± 0.007	0.012 (0.009–0.016) ^c^	0.008 (0.004–0.012) ^c^

Notes: ^†^ Model was unadjusted for any confounding variables. ^‡^ Multiple logistic regression model was used, while adjusting for gender, preterm birth, birth asphyxiation, fetal growth restriction, family income, paternal and maternal education, paternal and maternal age at childbirth. **^§^** Hierarchical linear regression model was used, while adjusting for gender, preterm birth, birth asphyxiation, fetal growth restriction, family income, paternal and maternal education, paternal and maternal age at childbirth. **^¶^** β was the coefficient for ETS exposure variable in the hierarchical linear regression model, where the dependent variable took the form of log (hyperactivity score + 1). ^a^ *p* < 0.05; ^b^ *p* < 0.005; ^c^ *p* < 0.001.
